# The Potential of Glycyrrhiza from “Medicine Food Homology” in the Fight against Digestive System Tumors

**DOI:** 10.3390/molecules28237719

**Published:** 2023-11-22

**Authors:** Doudou Lu, Yating Yang, Yuhua Du, Lei Zhang, Yi Yang, Joanna Japhet Tibenda, Yi Nan, Ling Yuan

**Affiliations:** 1 School of Clinical Medicine, Ningxia Medical University, Yinchuan 750004, China; ludoudou2022@163.com; 2 Traditional Chinese Medicine College, Ningxia Medical University, Yinchuan 750004, China; 20210410204@nxmu.edu.cn; 3 College of Pharmacy, Ningxia Medical University, Yinchuan 750004, China; 20210710269@nxmu.edu.cn (Y.D.); joannajaphet14@gmail.com (J.J.T.); 4 Key Laboratory of Hui Ethnic Medicine Modernization of Ministry of Education, Ningxia Medical University, Yinchuan 750004, China; 20160104@nxmu.edu.cn; 5 School of Basic Medical Sciences, Ningxia Medical University, Yinchuan 750004, China; 220220735@nxmu.edu.cn

**Keywords:** glycyrrhiza, natural distribution, digestive system cancer, pharmacology, medicine food homology, anti-tumor

## Abstract

Glycyrrhiza has a long history of applications and a wide range of pharmacological effects. It is known as the “king of all herbs”. Glycyrrhiza is effective in clearing heat, detoxifying, relieving cough, and tonifying qi and has good bioactivity in multiple inflammatory, immune, and tumor diseases. This review aims to summarize the origin, distribution, and anti-digestive system tumor mechanism of glycyrrhiza and its homologous applications in medicine and food. The active compounds include triterpenoids, flavonoids, and coumarins, which are widely used in clinical treatments, disease prevention, and daily foods because of their “enhancement of efficacy” and “reduction of toxicity” against digestive system tumors. This paper reviews the use of glycyrrhiza in digestive system tumors and provides an outlook on future research and clinical applications.

## 1. Introduction

Tumors remain one of the leading causes of mortality worldwide, and the dangers of digestive tumors should not be underestimated. Tumors of the digestive system are those which exist between the esophagus to the anal canal and include tumors of the esophagus, stomach, liver, gallbladder, pancreas, colorectum, etc. [1,2]. In the 2020 Global Cancer Epidemiology Survey, the top two countries in the incidence or mortality caused by cancer account for about 61% [3], showing that they seriously endanger human life and health while putting enormous economic pressure on society. Currently, the treatment of gastrointestinal tumors is related to the diagnostic grading and clinical stage of the disease, among other factors. Surgery combined with lymphadenopathy can be used at an early stage to inhibit the progression of the tumor, but due to the state of diagnostic techniques, economic capacity, and disease awareness rates, the first diagnosis in most patients is made much later than the appearance of clinical symptoms, which is one of the main reasons for missing out on the best surgical options. Therefore, chemotherapy has become the dominant measure for intermediate- and late-stage tumors. Although chemoradiotherapy has an inhibitory effect on some tumors, due to the toxic and side effects of drugs, it affects all organ systems and may cause acute and permanent damage, as well as common symptoms, including nausea, vomiting, diarrhea, severe anemia, neurotoxicity, etc. Many patients are intolerant of adverse reactions, leading to interruption of treatment and affecting the efficacy [4,5,6,7]. Therefore, it is of great clinical importance to explore new therapeutic modalities.

**Figure 1:**
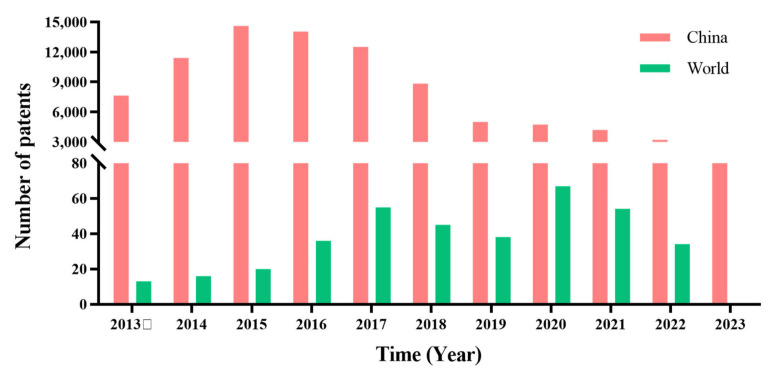
Number of glycyrrhiza patent applications from 2013 to 2023.

Nature is a treasure trove of thousands of medicines, including plants, ores, and animals, among which natural medicines have played an important role in healing since ancient times. Studies have shown that about 75 percent of the compounds in anti-cancer drugs, such as the familiar Vincristine and Paclitaxel, are derived from natural plants [8]. Glycyrrhiza is a perennial herb that grows all over the world. It is a common herbal plant in China and is known as the “king of herbs”. Since 25 AD, there has been a record of glycyrrhiza, which was put in the top category of the book named “*Shennong Herbal Classics*” [9]. In 2002, China’s National Health Commission issued the “Medicine Food Homology” (http://www.nhc.gov.cn/wjw/gfxwj/201304/e33435ce0d894051b15490aa3219cdc4.shtml accessed on 12 June 2023) catalog, and glycyrrhiza was also included in it. As a natural plant of “medicinal and food” origin, glycyrrhiza not only plays a pharmacological role in clinical practice, such as tonifying the qi and strengthening the spleen, relieving cough and phlegm, and detoxifying the body, but it also serves as an ingredient in daily foods and products, such as chewing gum, drinks, chocolate, tobacco [10], and whitening cosmetics. Because of its fatty roots and sweet juice, it is one of the most common drugs used in pediatrics to improve the bitter taste of Chinese medicine, which is also an important step to improve patients’ compliance with medical advice. There are currently about 79,345 patents related to glycyrrhiza as an ingredient, and about 2368 proprietary Chinese medicines and 279 health products have been developed, as shown in Figure 1.

**Figure 2:**
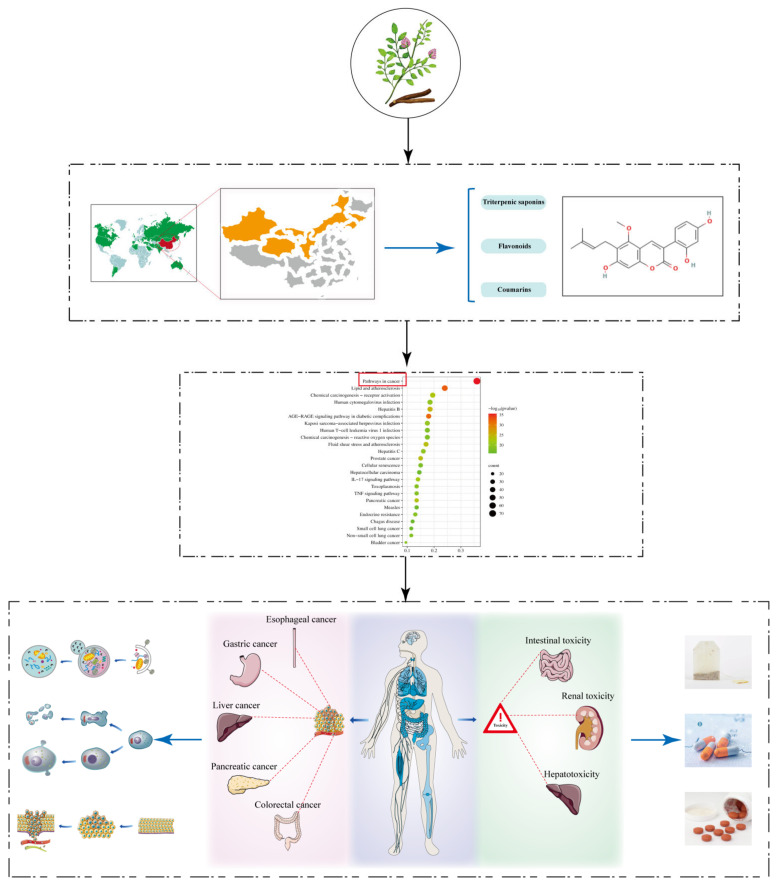
The research process of this review.

Due to the limitations of traditional pharmacological assays, the complex composition, numerous targets, and unclear mechanisms of natural drugs have been stumbling blocks for in-depth studies, but the new rise of mass spectrometry and purification and extraction techniques offers a convenient way to unravel the mysteries of the mechanism of action of natural drugs. Glycyrrhiza’s active ingredients are complex, with about 400 compounds obtained from the roots, which are classified as flavonoids, therapeutic agents, and coumarins, including glycyrrhizic acid, glycyrrhetinic acid, liquiritin, liquiritigenin, licoricchalcone, glycycoumarin, and others. These active ingredients exert a wide range of pharmacological effects, such as anti-inflammatory, anti-viral, antioxidant, and immune-modulating effects, among which anti-tumor effects should not be underestimated. As a natural plant of “medicinal food” origin, glycyrrhiza is both a “sensitizer” for chemotherapy drugs, for example by enhancing the efficacy of cisplatin and 5-fluorouracil, and an “attenuator” for radiotherapy modalities, improving liver damage, reducing nephrotoxicity, and alleviating enterotoxicity. A comprehensive review of the effect of glycyrrhiza on digestive tumors is still lacking, so this paper is a summary. A flowchart of the full paper is shown in Figure 2.

## 2. Origin of Glycyrrhiza

The word “glycyrrhiza” can be divided into two parts: “glycy-” is derived from the ancient Greek word “Glykos”, which means “sweet”, and “-rhiza” refers to the root of the plant; therefore, “glycyrrhiza” refers to a plant with a sweet root [11]. In China, we call it “Gancao”, as “Gan” also stands for sweetness, which is consistent with the ancient Greek symbolism. In Ayurvedic medicine, it is also known as “Rasayana”, which stands for a plant with nutritive and healing properties. 

**Figure 3:**
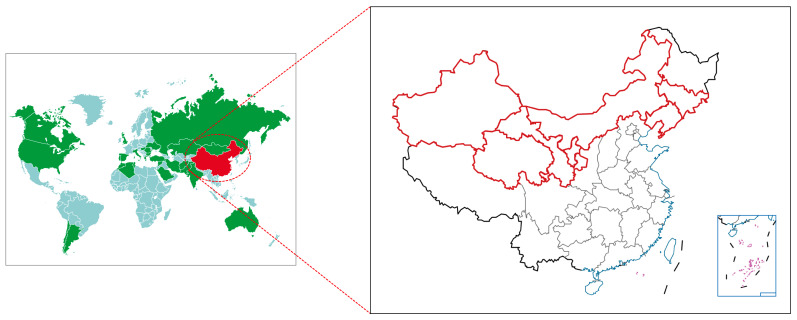
Distribution of glycyrrhiza in world and China. Green areas represent areas where three kinds of glycyrrhiza are grown, red represents China, and red lines represents areas where three kinds of glycyrrhiza are grown in China.

Glycyrrhiza belongs to a diverse genus of plants, and 29 species and six varieties have been reported [12]. Among them, three species are used as medicinal herbs, namely *G. glabra* L., *G. uralensis* Fisch., and *G. inflata* Batalin [13]. *G. glabra* L., known as “European glycyrrhiza”, is mainly sourced from regions such as Afghanistan, Syria, Persia, Southern Europe, and North Africa. *G. uralensis* Fisch. comes mainly from Siberia, China, and Mongolia. *G. inflata* Batalin, also known as Chinese glycyrrhiza, accounts for about 60% of the total wild-type glycyrrhiza in China [12]. All three species are cultivated in China, where they are mostly found on the northern border of Xinjiang, the Hexi Corridor in Gansu, the Ordos Plateau, the Northeast Horqin Steppe, and the Qinghai Plateau [14]. Figure 3 shows a map of the distribution of glycyrrhiza in the world and China.

## 3. The Active Ingredient of Glycyrrhiza

**Figure 4:**
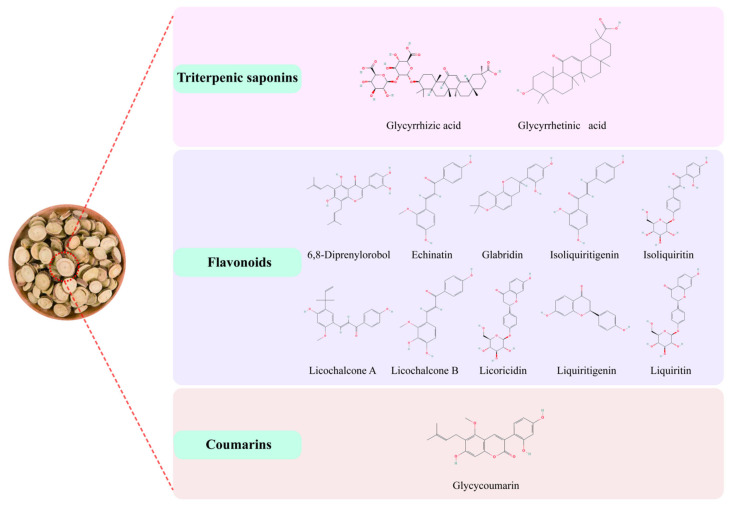
The active compounds in glycyrrhiza.

The three classes of compounds currently isolated and obtained from glycyrrhiza do not contain the same chemical compositions or contents due to differences in plant species, growing conditions, and processing methods [15]. Basic information on the active ingredients of glycyrrhiza is given in Figure 4 and Table 1.

### 3.1. Triterpenic Saponins

There are about 60 triterpenic saponins isolated from Glycyrrhiza glabra. These saponins are ingredients with the basic structure of 3β-hydroxy pentacyclic triterpene oleanol, which is also referred to as a derivative of β-coumarinol, mainly including glycyrrhizic acid and glycyrrhetinic acid. Glycyrrhizic acid is found in the roots of the plant in the form of calcium or potassium salts and is one of the main sources of sweetness produced by the plant [16]. Glycyrrhizic acid is one of the most important indicators to assess the quality of glycyrrhizin and should not be less than 2%. However, the oral bioavailability of glycyrrhizin acid is low at approximately 19.62%, which greatly limits its application. It has been shown that glycyrrhizic acid is the principal ingredient in the action of triterpenoids, and that the conversion of glycyrrhizic acid to glycyrrhetinic acid takes place as follows: glycyrrhizic acid is absorbed into the blood and reaches the small intestine, where it is hydrolyzed by β-glucuronidase to glycyrrhetinic acid by the action of intestinal flora to exert pharmacological effects [17].

### 3.2. Flavonoids

Flavonoids are a class of secondary metabolites that are widely distributed in nature [18], with about 300 species in glycyrrhiza. All of these compounds contain the parent nucleus (C6–C3–C6) of 2-phenyl chromogenic ketones [19], including isoflavones, chalcone, liquiritin, liquiritigenin, and glabridin. Among these, glabridin and glycyrrhiza chalcone A are species-specific compounds in *G. glabra* L. and *G. inflata* Batalin, respectively [20,21]. These ingredients are absorbed and metabolized by the liver and intestine to produce water-soluble products or glycosides, which are then excreted via the kidneys [22].

### 3.3. Coumarins

Coumarins are found mainly in *G. uralensis* Fisch. and have a similar structure to flavonoids, with a C6–C3–C6 basic skeleton [23], except that in coumarins, the carbonyl group is replaced by a benzene ring in an adjacent position, or the benzene ring and the parent nucleus can form a furan ring to form a coumestan (II) type structure [22], which has good biological activity.

**Table 1:** Basic information of active Compound in Glycyrrhiza.

Class	Compound	Molecular Formula	CAS	OB (%)	Species			Ref.
					*G. glabra* L.	*G. uralensis* Fisch.	*G. inflata* Batalin	
Triterpenic Saponins	Glycyrrhizic Acid	C_42_H_62_O_16_	1405-86-3	19.62	+	+	+	[24]
	Glycyrrhetinic Acid	C_30_H_46_O_4_	471-53-4	22.05	+	+	+	[25,26]
Flavonoids	6,8- Diprenylorobol	C_25_H_26_O_6_	66777-70-6	1.22	−	+	−	[27]
	Echinatin	C_16_H_14_O_4_	34221-41-5	66.58	+	+	+	[24,25]
	Glabridin	C_20_H_20_O_4_	59870-68-7	53.25	+	−	−	[28]
	Isoliquiritigenin	C_15_H_12_O_4_	961-29-5	85.32	+	+	+	[29]
	Isoliquiritin	C_21_H_22_O_9_	5041-81-6	8.61	+	+	+	[30,31]
	Licochalcone A	C_21_H_22_O_4_	58749-22-7	40.79	+	−	+	[30,32,33]
	Licochalcone B	C_16_H_14_O_5_	58749-23-8	76.76	+	+	+	[24,34,35]
	Licoricidin	C_26_H_32_O_5_	30508-27-1	0.99	+	+	−	[36]
	Liquiritigenin	C_15_H_12_O_4_	578-86-9	32.76	+	+	+	[29]
	Liquiritin	C_21_H_22_O_9_	551-15-5	65.69	+	+	+	[30]
Coumarins	Glycycoumarin	C_21_H_20_O_6_	94805-82-0	23.56	−	+	−	[37]

CAS: The CAS number is a unique numerical identification number for a chemical substance.

## 4. Mechanism of Action

**Figure 5:**
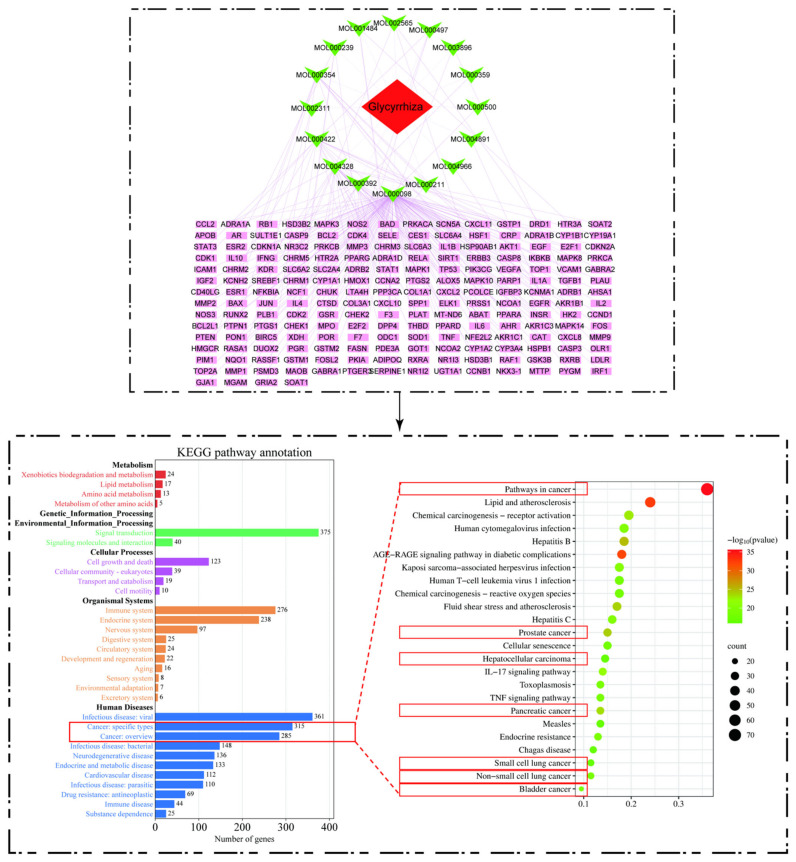
Screening of the related targets and pathways of glycyrrhiza.

**Figure 6:**
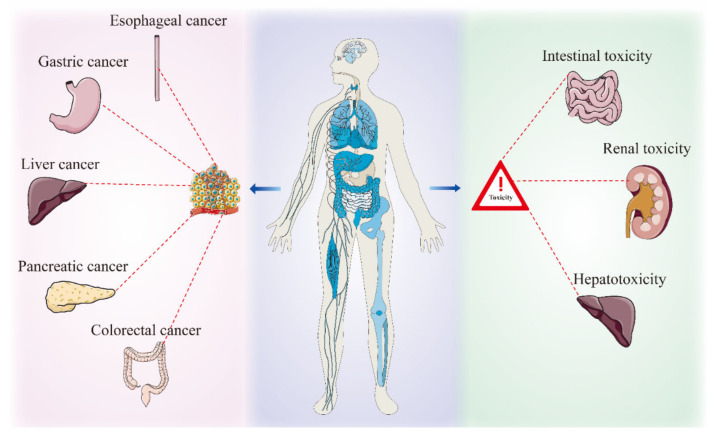
Synergistic and attenuated effects of glycyrrhiza on digestive tumors.

Natural plant-based medicines are characterized by complex compositions, diverse targets, and unknown mechanisms of action, which cannot be comprehensively studied and explored by conventional pharmacological methods. With the new emergence of the discipline of “pharmacology”, the research of natural plants has been fast-tracked and their mysteries are expected to be unveiled. Network pharmacology is a new discipline that uses network analysis of biological systems to select specific signaling nodes for the design of multi-target drug molecules [38]. We used the Traditional Chinese Medicine Systems Pharmacology Database and Analysis Platform (TCMSP, https://old.tcmsp-e.com/tcmsp.php (accessed on 15 June 2023)) to search for potential targets of active ingredients. This is a database containing information such as pharmacokinetics, potential targets, and diseases for a large number of compounds. We set the parameters to DL ≥ 0.18 and OB ≥ 30% and finally obtained 16 active ingredients and 200 action targets. To investigate the effects of glycyrrhiza targets on diseases and physiopathology, 200 targets were enriched and analyzed using the STRING database (https://cn.string-db.org/ (accessed on 15 June 2023)), which can analyze protein–protein interactions. The results showed that the key active ingredient of glycyrrhiza had a pharmacological effect primarily on tumors, including prostate, liver, pancreatic, and lung cancers, as shown in Figure 5. Based on the enrichment results, we summarized the mechanisms of “potentiation” and “toxicity reduction” of glycyrrhiza active ingredients against tumors, as shown in Figure 6.

### 4.1. Potentiation of Tumors by Glycyrrhiza

**Figure 7:**
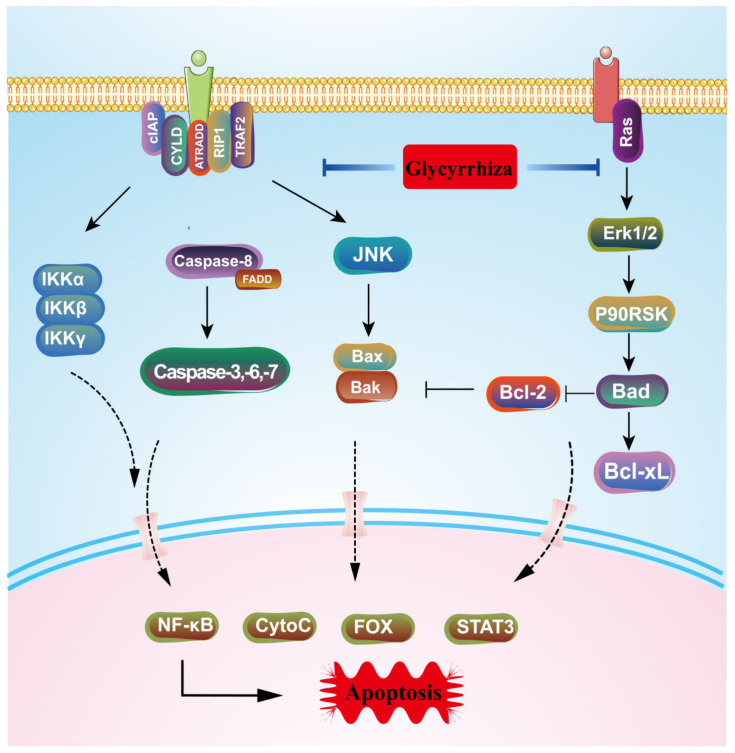
The process of apoptosis regulated by glycyrrhiza.

6,8-Diprenylolobol is a natural flavonoid with a variety of biological activities such as anti-fungal [39], anti-*Helicobacter pylori*, and anti-tumor effects [40] and is believed to be a natural chemotherapeutic agent against tumors [41], exerting anti-cancer effects primarily by inducing apoptosis. Apoptosis is a form of programmed cell death [42], a spontaneous “suicide” process that occurs when cells are stimulated by inflammatory factors, oxidative stress, etc., in order to maintain a stable living environment for the body. The nature of tumorigenesis is an uncontrolled balance between apoptosis and proliferation, and the induction of cell death to limit wireless proliferation is key to the effectiveness of treatment. Forkhead box O3 (FOXO3), a member of the O subclass of the forkhead family of transcription factors, induces cell death primarily by modulating the expression of a protein associated with apoptosis, and cellular DNA damage can activate FOXO3 expression [43]. Chang Min Lee [27] found that 6,8-diprenylorobol induced an increase in ROS levels; triggered the transcriptional activity of FOXO3; regulated the expression of Bax, Bim, p21, p27, Bcl-2, and Bcl-XL; and blocked the growth of Huh-7 and HepG2 cells. Yong Jun Choi et al. [44] reached the same conclusion for colorectal cancer. In SW480 cells, Xian Shao et al. [45] found that 6,8-diprenylorobol is involved in the activation of the Akt/mTOR signaling pathway and initiates the activation of caspase-3, a key protein in apoptosis, in addition to modulating FOXO3 transcription factor activity, and confirmed that the isoprenyl group is the dominant structural motif for the anti-tumor effect of this ingredient, as shown in Figure 7.

Echinatin was originally obtained in 1971 from isolated cell cultures by Furuya and other scientists, who showed that the ingredients induced apoptosis to act against esophageal and colorectal cancers. Since the discovery of platinum-based drugs in the 1960s, they have been widely used to treat a variety of tumors, with oxaliplatin, a third-generation platinum-based drug that targets DNA to cause damage and inhibit tumor cell proliferation, being one example, but the efficacy of this drug still needs to be improved due to increasing drug resistance [46]. Echinatin can be used to improve drug resistance and increase the sensitivity of chemotherapeutic drugs to cells. Ah-Won Kwak et al. [47] used oxaliplatin-susceptible and resistant tumor cells as a model and found that echinatin induced ROS-mediated apoptosis through the JNK/p38 MAPK signaling pathway after administration of 5–15 µm doses and still showed good anti-cancer activity against tumor cells of resistant strains. In addition, the team reached the same conclusion in a model of esophageal cancer cells [48]. Pan Hong et al. [49] used 20–50 mg/kg of echinatin to intervene in KYSE270 xenograft model nude mice and achieved a 48–57% reduction in tumor growth with no significant effect on glutamate transaminase (ALT) or aspartate transaminase (AST) in mice, suggesting that echinatin can exert anti-cancer activity with minimal liver and kidney damage.

Glabridin, mainly produced by the genus *Glycyrrhiza glabra* L., was first isolated from the rhizome of Glycyrrhiza glabra by Japanese scientists, and its content accounts for about 11 percent of the total flavonoid content. Invasion and metastasis, among the 14 important tumor phenotypes identified by Douglas Hanahan [50], are important factors in promoting tumor progression and are among the leading causes of clinical treatment failure. Important proteins involved in this process, known as MMPs or matrix metalloproteins, show high expression in a variety of malignancies, including breast, oral, and lung cancers, and can therefore serve as important markers of tumor pathology and aggressiveness [50]. In the early stage of disease, highly expressed MMP-9 can produce a variety of membrane growth factors to create a suitable environment for tumorigenesis, followed by the stimulation of host cells by MMP-inhibitors to produce large amounts of MMP-9, which are absorbed and used by tumor cells to synthesize nutrients for their own growth. In the middle and late stages of tumor development, MMP-9 can also promote the expression of VEGF to promote invasion and metastasis in blood vessels [51]. Tingting L [52] found that the PI3K/Akt signaling pathway was activated after 40 μg/mL of glabridin intervention in colorectal cancer cells, and the downstream transcription factor mTOR was able to suppress the transcription of MMP-9 and play an inhibitory role in metastasis. The disruption of the basement membrane is the key step in invasion and requires the activation of MMPs. Inhibition of the activation or expression of MMPs may shed new light on the treatment of tumor metastasis. The promoter region of the MMP gene shows significant conservation of regulatory elements such as AP-1, NF-κB, and SP-1 [53,54], while the activation of MMP9 is regulated by AP-1 and NF-κB. Ming-Ju Hsieh [55] demonstrated that glabridin inhibited the DNA-binding activity of NF-κB and AP-1, as well as inhibiting i-κB α phosphorylation and decreased the expression of c-Jun and c-Fos, resulting in the down-regulation of MMP9, which had certain inhibitory effects on the metastasis and invasion of hepatocellular carcinoma.

Isoliquiritigenin levels are low in glycyrrhiza, accounting for an average of seven parts per million of the total composition of glycyrrhiza, but this compound has high biological activity, with an oral bioavailability of 85.32% in vivo, and has inhibitory effects on a variety of tumors such as liver cancer, pancreatic cancer, and esophageal cancer. The cell cycle is a repetitive biological process that begins at the end of the last stage of mitosis and ends at the end of the next stage, and it is important for cell proliferation and damage repair [56]. This process is finely and rigorously regulated by a variety of proteins, kinases, and transcription factors in the organism, the main network of which is the cyclic phosphorylation and dephosphorylation of the cyclin–CDK axis. The activation of CDKs is regulated by cyclins. Cyclin binds to CDK and assembles into a kinase-active complex that regulates the downstream transcription factor E2F, promotes the expression of protein genes involved in DNA synthesis, and drives the cell cycle in an ordered fashion. The disruption of this process is one of the characteristic manifestations of malignant tumors. Lei Song et al. [57] found that isoliquiritigenin reversed the inhibitory effect of LY294002 on the PI3K/Akt signaling pathway, reducing activation of mTOR active sites, up-regulating Beclin-1 expression, and inhibiting hepatocellular carcinoma growth. The same results were obtained for stomach cancer [58]. In addition, isoliquiritigenin mediates the down-regulation of cyclin D1 by blocking the activity of AP-1 transcription factors in the G0/G1 phase of the cell cycle [59,60]. It also modulates the up-regulation of the negative regulatory proteins P21 and P27 and the down-regulation of cyclin D1 expression, and it induces cell arrest in the G1/S phase by a mechanism likely involving PI3K/Akt activation.

**Figure 8:**
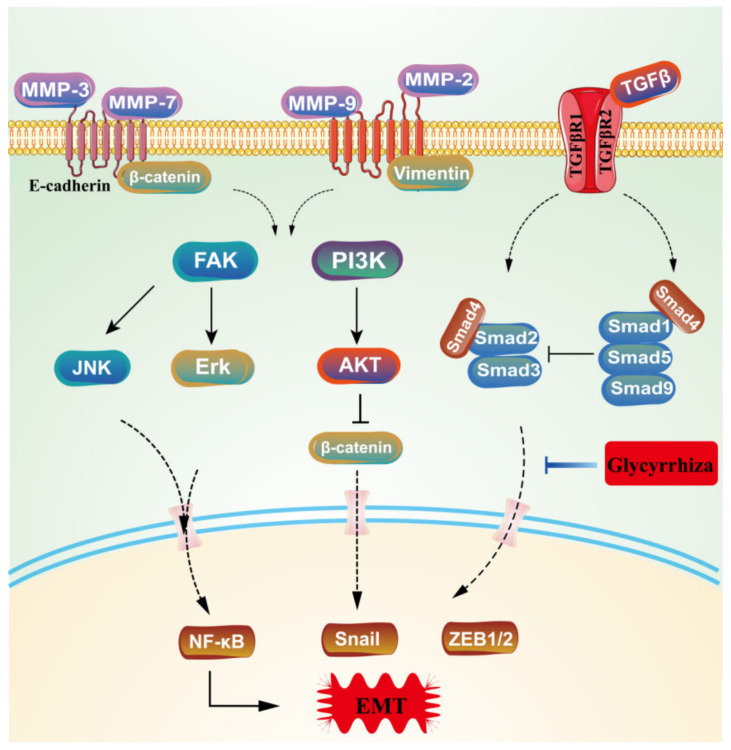
The process of EMT regulated by glycyrrhiza.

Both liquiritin and liquiritigenin are flavonoids that regulate cellular EMT processes and inhibit tumor invasion and metastasis. This process is a key step in the progression of tumor invasion and can confer greater ability to invade tumor cells. The activation of EMT requires the activation and regulation of a wide range of genes and transcription factors, including N-cad, E-cad, ZEB1, Snail1, and Vim [61]. Fan-Chun Meng et al. [62] proposed that liquiritigenin reverses Runx2-induced EMT, reducing the expression of E-cad and up-regulated N-cad, presumably through Runx2-mediated inactivation of the PI3K/Akt pathway. Another study confirmed that TRAIL, in combination with liraglutin E-cadherin, enhances the expression of N-cadherin, Vimentin, and Twist, which are characteristic markers of mesenchymal cells [63], as shown in Figure 8.

Licoflavone A has been shown to have good inhibitory effects on gastric and hepatocellular carcinomas, modulating cell proliferation, apoptosis, and metastasis processes. An important target is VEGF, vascular endothelial growth factor, which is thought to act as a switch for angiogenesis, and the activation and deactivation of this gene can regulate vascular permeability and cell migration processes [64]. Wenjin Hao et al. [65] used 100 μM of licoflavone A to intervene in BGC and GES-1 cells and found that the drug decreased the viability of gastric cancer cells by 66.67% but had no significant effect on normal cells, suggesting that the drug has a good safety profile. It has also been shown to inhibit tumor cell proliferation, invasion, and metastasis by modulating the Ras/Raf/MEK and PI3K/Akt signaling pathways, which mediate a reduction in the expression of MMP2, MMP9, N-cadherin, Bcl-2, caspase, and other related proteins [66,67,68,69].

Glycycoumarin has well-established biological effects in vivo and exhibits anti-tumor activity primarily by targeting survivin [64]. Survivin, also known as BIRC5, is the smallest member of the IAP family of inhibitors of apoptosis proteins and has the structural domain of a baculovirus repeat sequence (BIR) that binds to E3 ubiquitination ligases, ubiquitin-related (UBA) structural domains, ubiquitin-coupled (UBC) structural domains, and caspase-recruitment domains (CARD). It has been shown that this protein is located at the center of tumorigenesis and can be regulated by upstream kinases and transcription factors, as well as negatively regulating the expression of upstream molecules, forming a bidirectional regulatory network. Survivin is highly expressed in a variety of malignancies, and SurVaxM, the world’s first peptide mimetic oncology vaccine, works by stimulating T- and B-lymphocytes to target and inhibit survivin in tumor tissue where it is highly expressed. Enxiang Zhang et al. [70] demonstrated that in HepG2 cells, glycycoumarin in combination with ABT-737 or orlistat significantly reduced survivin expression and may be associated with increased sensitivity to the drug ABT-737. In contrast, xenograft models have been shown to reduce tumors by up to three-fold. It has been suggested that the drug could be used as a chemotherapeutic booster to increase the targeting effect on survivin.

**Figure 9:**
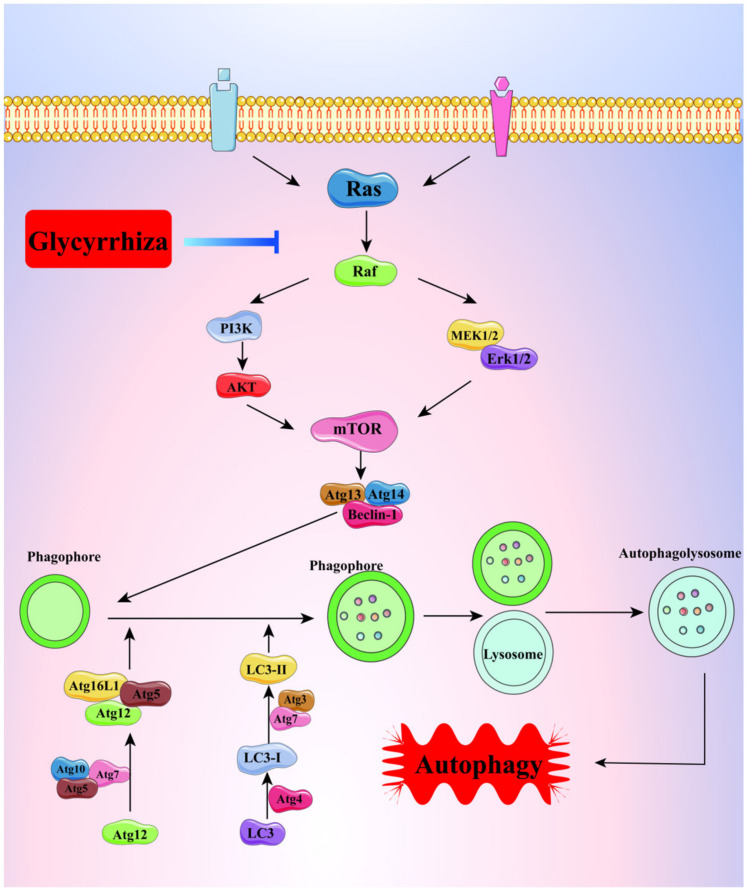
The process of autophagy regulated by glycyrrhiza.

Glycyrrhizic acid and glycyrrhetinic acid are both triterpenoids, with glycyrrhetinic acid playing a major role in exerting pharmacological effects. Autophagy, a common way for tumor cells to escape and resist death, has a double-edged effect. In environments such as hypoxia and starvation, protective autophagy can be triggered to speed up the process of material and energy recycling and metabolism through the action of lysosomes, helping the cell survive dangerous periods; however, over-activated autophagy can inhibit tumor growth, so this process is also known as “programmed death type II” [71]. During autophagy, LC3 is sheared, processed, and modified by a ubiquitin-like system under the action of Atg7 and Atg3 molecules to form LC3-II in lipid form and bind to the bilayer membrane of autophagic lysosomes, so LC3-II is regarded as an important molecular marker of autophagy, and its content is proportional to the degree of autophagy. Both glycyrrhizic acid and glycyrrhetinic acid were shown to induce autophagy in hepatocellular carcinoma cells, and the mechanism of action may be related to the up-regulation of p-ERK1/2 expression and the promotion of LC3-II binding in autophagic lysosomal membranes [72,73], as shown in Figure 9.

Licochalcone B is a flavonoid that can have anticancer effects by inducing hepatocellular carcinoma cells to stall in the G2/M phase. The cell-cycle checkpoint is a system of mechanisms that self-check DNA and chromosomes, and if DNA is damaged during the cell cycle, the checkpoint system is quickly activated to block the continuation of the cycle. The G2/M phase is primarily concerned with preventing the introduction of damaged DNA genomes into the next mitotic step, whereas cyclin B1, CDK1, and CHK2 are important for regulating the G2 transition. The phosphorylation of CHK2 initiates the G2/M phase checkpoint, and CDK1 and cyclin B1 promote the expression of the morphogen MPF during cell-cycle progression towards the M phase. Previous studies confirmed that site B down-regulates the expression of cyclin B1, CDK1, and CHK2 in hepatocellular carcinoma cells at doses ranging from 10 to 120 um and induces cell clumping in the G2/M phase [74,75]. In contrast, the G1/S cell-cycle checkpoint, which regulates the entry of cells into the S phase of DNA synthesis through the G1 phase, is primarily affected by CDK4D and cyclin D. Licoricidin can induce tumor cell-cycle arrest in the G0/G1 phase in a concentration-dependent manner, which may be related to the regulation of ICMT/Ras processes [76], as shown in Table 2.

### 4.2. Attenuation of Tumors

Anti-tumor drugs such as platinum, fluorouracil, methotrexate, and cyclophosphamide target DNA or RNA and interfere with their synthesis or transcription processes to exert anti-tumor effects [46,82,83]. However, due to poor tissue- and organ-specific inhibition, these drugs tend to accumulate in certain local organs, such as the heart, kidney, and bone marrow, while exerting pharmacological effects. After the drug is absorbed, its metabolites are mainly excreted by the liver and kidneys, so long-term use can easily increase the burden on metabolic organs, which in turn can hinder the absorption and metabolism of the drug, creating a vicious cycle. Natural drugs play an important role in clinical practice. In addition to enhancing the sensitivity and targeting of radiotherapy drugs, they can also reduce the cytotoxic effects of drugs, such as enterotoxicity, hepatotoxicity, and nephrotoxicity, and play a role in toxicity reduction.

Intracellular toxicity is one of the main reasons for the decreased efficacy of antineoplastic drugs. In a physiological state, the small intestinal mucosa, consisting of crypts and villi, increases the area available for absorption and metabolism, and the structural integrity of the intestinal mucosa depends on the rate of crypt cell production and the crypt/villi ratio. Chemotherapeutic agents induce the reduction of the villous region, distortion of the mucosal glandular structure, crypt ablation, cytoplasmic vacuolization, and finally acute necrosis, where the loss of the regenerative capacity of the intestinal mucosal crypt due to cisplatin is permanently irreversible. Clinical symptoms of chemotherapy, such as gastrointestinal dysfunction, diarrhea, delayed gastric motility, mucositis, and impaired barrier function, may persist long after the completion of the treatment regimen [76]. Studies have shown that 70–80% of patients experience nausea and vomiting, and about 67% experience diarrhea. When combined with neutropenia, death may result from severe infection. Summya Rashid et al. [84] used cisplatin to successfully induce intestinal toxicity in an animal model of rats, and the administration of 18β glycrrhetinic acid resulted in decreased expression of MAD, TNF-α, and NF-κB and increased glutathione levels, suggesting that this compound could reduce the inflammatory response and enhance the antioxidant capacity of the body. It has been shown that the non-selective cytotoxicity of 5-FU induces ROS production; promotes DNA damage; continues to activate NF-κB [85]; leads to the release of inflammatory mediators such as TNFα, COX-2, iNOS, and IL-1β [86]; and amplifies the signals and production of inflammatory mediators. In contrast, 6-isoliquiritigenin reverses this process [87], offering a new option for the treatment of chemotherapy-associated enteritis.

Drug-related hepatotoxicity is one of the most common clinical causes of discontinuation [88]. Peroxisome proliferator-activated receptor (PPARg), a ligand-induced transcription factor, plays an important role in the protection against drug-induced liver injury. Activated PPARg binds to specific regions of the target gene and regulates gene expression. Methotrexate disrupts antioxidant defense mechanisms and stimulates the production of inflammatory factors, leading to cytotoxic effects in the liver. Ayman M Mahmoud et al. [89] demonstrated that 18β-GA intervention in MTX-induced liver injury rats significantly improved the levels of ALT, AST, ALP, LDH, GGT, and total bilirubin in peripheral serum and restored the liver’s ability to synthesize protein. Yuzhu Cao et al. [90] demonstrated the same hepatoprotective pharmacological effect of magnesium at 9 mg/kg and 18 mg/kg, with a mechanism likely involving the co-localized expression of nodal COX-2, ZO-1, and Cx43. Prerna Chauhan et al. [91] found that MTX induced pathological manifestations such as cytoarchitectonic disruption, sinusoidal dilatation, hemorrhage, congestion, and nuclear protrusion in rat livers, while glycyrrhizin at 400 mg/kg had the greatest protective effect. Regulatory T cells play a key role in immune tolerance and inhibition of anti-tumor responses. Xu-ying Wan et al. [91] demonstrated that glycyrrhizin increased the CD4^+^/CD8^+^ ratio of peripheral leukocytes in mice, enhancing the protective effect on the liver. The combined application of LPS-GalN is a well-established method to cause liver injury in animal models and has been widely used in experimental studies of liver injury. Hongming Lv et al. [1] proposed that the mechanism by which licochalcone A exerts its hepatoprotective effect is associated with the inhibition of lps-induced activation of the inflammatory response by NF-κB and p38/ERK MAPK signaling pathways, in addition to the promotion of Atg7-, Atg5-, Beclin-1-, Atg5-, Atg3-, and LC3II-induced autophagy activation by promoting the protein conversion levels of Atg7, Atg5, Atg3, and LC3II.

The kidney is a very important excretory organ, and many drugs are absorbed and metabolized and then excreted from the body in the form of metabolites, preventing poisons and other toxins from accumulating in the body. The kidney is a major target of cisplatin metabolism, and approximately 20% of patients develop severe renal dysfunction during treatment. Studies have shown that cisplatin concentrations in proximal tubular epithelial cells are approximately five times higher than those in serum concentrations [92]. Wani Arjumand et al. [93] found that glycyrrhizic acid restored serum levels of BUN, creatinine, and LDH and reversed pathological manifestations of tubular and glomerular congestion, edema, and necrosis. Apoptosis plays an important role in cisplatin-induced nephrotoxicity, and an imbalance in the ratio of Bax to Bcl-2 is one of the most important causes of induced renal cell death. Chi-Hao Wu et al. [94] found that glycyrrhizic acid and 18β-glycyrrhetinic acid exerted a mechanism to protect against renal function, probably by reducing the production of pro-inflammatory factors HMGB1, NF-κB, TNF-α, and IL-1β. Zhiyin Pei et al. [95] suggested that isoliquiritin reduces Bax protein levels in cisplatin-induced cells, increases Bcl-2 expression, and reduces cisplatin-induced apoptosis in renal tubular epithelial cells, as shown in Table 3.

## 5. Health Food

Glycyrrhiza, an herbal medicine, has been made into a variety of health foods due to its rich efficacy and high safety profile, mainly related to immune regulation, liver function protection, and throat clearing, as shown in Table 4.

## 6. Conclusions and Outlook

Glycyrrhiza glabra is a widely used natural plant, and more than 400 high-quality articles on glycyrrhiza glabra have been published in the last decade, comprehensively describing its applications in cardiovascular disease, infectious diseases, liver disease, and oncology and demonstrating the pharmacological effects of glycyrrhiza glabra and its active ingredients in antioxidant, anti-inflammatory, and anti-viral applications. However, a comprehensive review of glycyrrhiza glabra active ingredients against digestive tumors is lacking. In this paper, we review the “potentiation” and “toxicity reduction” of triterpenoids, flavonoids, and coumarins, the active components of glycyrrhiza, in the treatment of digestive tumors. In terms of “potentiation”, the active ingredients of glycyrrhiza, such as glycyrrhizic acid, glycyrrhetinic acid, liquiritin, liquiritigenin, and licoricchalcone, have shown good biological activities against various gastrointestinal tumors such as esophageal cancer, gastric cancer, liver cancer, pancreatic cancer, and colorectal cancer, as they can induce apoptosis, autophagy, block cell cycling, and reduce metastasis, among other effects. The mechanism involves the regulation of JNK/p38 MAPK, PI3K/Akt/mTOR, MAPK/STAT3/NF-κB, and other signaling pathways, which affect the regulation of related proteins via transcription factors. In terms of “toxicity reduction”, glycyrrhiza also plays an important role in improving the cytotoxicity caused by radiotherapy drugs by reducing intestinal mucosal damage, improving hepatotoxicity, and restoring kidney function. As an important ingredient in medicinal foods, glycyrrhiza is made into a variety of health products that, among other things, help tumor patients improve their immunity and prevent chemical liver damage. Based on the “potentiation” and “attenuating” effects of glycyrrhiza on digestive tract tumors, in vitro cellular experiments and in vivo animal experiments are still the main focus, and there is a lack of clinical correlation tests, which may be the next focus of in-depth research on the anti-tumor effects of glycyrrhiza.

## Figures and Tables

**Table 2:** Types and mechanisms of cancer treatment with glycyrrhiza.

Compound	Cancer	Experimental Model	Dosage (μM)	Mechanism	Phenotype	Ref.
6,8- Diprenylorobol	Liver Cancer	HepG2, Huh-7	20–60	↑PARP1, ↑Caspase-3, ↑FOXO3, ↑Bax, ↑Bim, ↑p21, ↑p27 ↓Bcl-2, Bcl-XL	Proliferation Apoptosis	[27]
	Colorectal Cancer	LoVo, HCT15, HCT29, NCI-H508, HCT8, DLD1, CCD18-Co	5–60	↑FOXO3, ↑p53, ↑p27, ↑p21, ↑p-Ser15, ↑p-Ser20, ↑p-Ser46 ↓Akt, ↓ERK, ↓JNK, ↓p38, ↓ROS	Proliferation Apoptosis	[44]
	Colorectal Cancer	SW480 cells	5–20	↑Caspase-3, ↑LC3-II/LC3-I, ↑PARP-1 ↓p62, ↓p-Akt, ↓p-mTOR	Proliferation Apoptosis Autophagy	[45]
Echinatin	Esophageal Cancer	KYSE30, KYSE270	10–40	↑LC3, ↑Caspase-3, ↑PARP ↓p-Akt, ↓p-mTOR	Proliferation Apoptosis Autophagy	[49]
		KYSE 30, KTYSE 70, KTSE 410, KYSE 450, KYSE 510	5–15	↑DR4, ↑DR5, ↑CHOP, ↑GRF78, ↑Bax, ↑p-JNK, ↑p-p38, ↑Apaf-1, ↑c-PARP ↑ADP-Ribose ↓Bid, ↓Bcl-2	Proliferation Apoptosis Cell Cycle (G2/M)	[48]
	Colorectal Cancer	HT116, HT29, HaCaT, JB6	5–15	↑ROS, ↑p-JNK, ↑p-p38, ↑Bim, ↑Bax, ↑Cyto C, ↑Aparf-1 ↓Mcl-1, ↓Bid, ↓Bcl-XL, ↓Caspase-3, ↓Bcl-2	Proliferation Apoptosis Cell Cycle (G2/M)	[47]
Glabridin	Colorectal Cancer	SW480, SW620, HT29, HCT116	5–40	↑Bax, ↑cleaved-Caspase-3/9 ↓Bcl-2, ↓MMP9, ↓PI3K, ↓Akt, ↓mTOR	Proliferation Apoptosis Migration Invasion	[52]
	Liver Cancer	Huh7, Sk-Hep-1	10–40	↓MMP9, ↓TIMP-1, ↓NF-κB, ↓AP-1, ↓c-Fos, ↓c-Jun, ↓p-IκB, ↓ERK1/2, ↓JNK1/2	Proliferation Apoptosis Migration Invasion	[55]
	Liver Cancer	Huh7, HepG2, Sk-Hep-1	25–100	↑Caspases-3/8/9, ↑PARP, ↑LC3, ↑p38, ↑Beclin-1, ↑JNK1/2, ↑ERK1/2	Proliferation Apoptosis Autophagy	[77]
Glycycoumarin	Liver Cancer	HepG2, Huh-7, SMMC-7721, HepG2 xenograft model	10–40	↓Survivin, ↓p-TOPK	Proliferation	[70]
Glycyrrhetinic Acid	Liver Cancer	HepG2, Hep3B	20–60	↑LDH, ↑Bax, ↑Caspase-3, ↑LC3-II, ↑p-ERK	Proliferation Autophagy Apoptosis	[72]
Glycyrrhizin	Liver Cancer	HepG2, MHCC97-H Nude mice	1–2 mmol/L	↑LC3-II, ↑p-ERK1/2 ↓AO, ↓p-P85, ↓p-Akt, ↓p-62P, ↓p-p70S6K, ↓Akt/mTOR	Proliferation Migration Autophagy	[73]
Isoliquiritigenin	Pancreatic Cancer	PANC1, MIA PaCa-2, hTERT-HPNE	10–25	↑LC3I/II, ↑p62	Proliferation Apoptosis Autophagy	[78]
	Liver Cancer	HCC97-H, LO2, SMMC7721, male BALB/c nude mice	12.5–50	↑Bax, ↑cleaved-Caspase-3, ↑P62 ↑LC3-II, ↑Beclin-1, ↑cleaved-PARP ↓Bcl-2, ↓p-Akt, ↓p-mTOR	Proliferation Apoptosis Autophagy	[57]
	Esophageal Cancer	KYSE140, KYSE520	5–20	↓EGFR, ↓Akt, ↓ERK1/2, ↓Jun, ↓Fos, ↓AP-1, ↓Cyclin D1	Proliferation	[60]
	Liver Cancer	Hep3B, LO2	40–50	↑P21,↑P27 ↓CyclinD1,↓Vimentin, ↓N-cadherin, ↓PI3K	Proliferation Migration Metastasis	[59]
	Gastric Cancer	SGC-7901, BGC-823, GES-1	20–40	↑LC3B II, ↑Beclin 1 ↓p62, ↓p-Akt, ↓p-mTOR	Apoptosis Autophagy Cell Cycle (G2/M)	[58]
	Liver Cancer	HepG2, Hep3B, L-02, QSG-7701	20–100	↑p21, ↑p-JNK, ↑p-p38, ↑IκB,↑ROS ↓p27, ↓Cyclin B1, ↓CDK1/2, ↓p-ERK, ↓p-STAT3, ↓STAT3, ↓p-IκB, ↓NF-κB (p65)	Apoptosis Cell Cycle (G2/M)	[79]
Liquiritin	Gastric Cancer	DDP-resistant SGC7901/DDP cells	20–80	↑p53, ↑p21, ↑Caspase-3/8/9, ↑PARP, ↑LC3B, ↑Beclin 1 ↓Cyclin D1, ↓Cyclin A, ↓CDK4	Proliferation Apoptosis Autophagy Cell Cycle (G0/G1)	[80]
	Gastric Cancer, Liver Cancer	AGS, SNU-216,GES-1, HL7702, HK2	50	↑Caspase-3/8/9,↑PARP, ↑Bax, ↑FADD, ↑DR4, ↑DR5, ↑ROS, ↑p-JNK ↓Bcl-2	Apoptosis EMT	[63]
Liquiritigenin	Liver Cancer	PLC/PRL/5, HepG2	200–400	↑p-JNK, ↑P38, ↑Caspase 3 ↓Bcl-2, ↓Bcl-xL, ↓PARP, ↓p-ERK, ↓ROS, ↓LDH	Apoptosis	[81]
	Colorectal Cancer	CRC HCT116 cells	10–100 μg/mL	↑N-cad, ↑Vim, ↑ZEB1, ↑Snail1 ↓PCNA, ↓Cyclin A, ↓Cyclin E1, ↓CDK2, ↓cyclin D1, ↓CDK4, ↓E-cad, ↓Runx2, ↓p-PI3K/PI3K, ↓p-Akt/Akt	Proliferation Invasion EMT	[62]
Licochalcone A	Gastric Cancer	BGC-823, GES-1	20–100	↑ROS, ↑Caspase-3, ↑PARP, ↑ERK, ↑JNK, ↑p38 MAPK	Proliferation Apoptosis	[65]
	Gastric Cancer	MKN45, SGC7901, GES-1	15–60	↓Glut1, ↓PKM2, ↓LDHA, ↓ERK, ↓Akt, ↓NF-κB, ↓PARP, ↓Caspase-3	Proliferation Apoptosis	[68]
	Liver Cancer	SK-Hep-1, HA22T/VGH	5–20	↓p-JNK1/2, ↓p-MKK4, ↓NF-kB(p65)	Migration Invasion	[67]
	Gastric Cancer	MKN-28, AGS, MKN-45, GES-1	5–50	↑Rb, ↑Bax, ↑PARP ↓CyclinA, ↓CyclinB, ↓MDM2, ↓Bcl-2, ↓Caspase-3	Apoptosis Cell Cycle (G0/G1)	[69]
	Liver Cancer	A549, HeLa, Hep3B	10–50	↑PARP, ↑Caspase-8 ↓PD-L1, ↓IκBα, ↓IKKα/β, ↓TRAF2, ↓RIP1, ↓Ras, ↓p-MEK, ↓p-Raf, ↓p65, ↓Ras	Proliferation Apoptosis	[66]
Licochalcone B	Liver Cancer	HepG2, Huh7	10–20	↑γ-H2AX foci, ↑p27, ↑ERK1/2, ↑JNK1/2, ↑p38 MAPK, ↑PARP ↓CyclinB1, ↓Cdc2, ↓p-Cdc2, ↓Akt, ↓p70S6K, ↓Cyclin D1	Proliferation Apoptosis Cell Cycle (G2/M)	[75]
	Liver Cancer	HepG2, HEK293, EAhy926	40–120	↑ZBTB17, ↑CDC20, ↑PKMYT1, ↑GADD45A, ↑GADD45B, ↑SFN, ↑CDKNIC, ↑p21, ↑p53, ↑TNF,↑TNF-R1, ↑Fas, ↑FasL, ↑JUN, ↑FOS, ↑TNF-R1, ↑Bid, ↑Bak, ↑PUMA, ↑ERN1, ↑LMNA, ↑DDIT3, ↑ROS, ↑DIABLO, ↑ENdoG, ↑Caspase -3/8/9 ↓CDK1, ↓CyclinB1, ↓CHK2, ↓CDC7, ↓Bcl-xl, ↓CDC14B, ↓PARP4, ↓BIRC3	Proliferation Apoptosis Cell Cycle (G2/M)	[74]
Licoricidin	Gastric Cancer	MGC-803	5–20	↑Bax, ↑Cyt-C, ↑Caspase-3 ↓Bcl-2, ↓CyclinD1, ↓CDK4, ↓MMP, ↓MMP9, ↓Ki-67, ↓ICMT, ↓Ras-GTP, ↓Raf, ↓Erk	Proliferation Cell Cycle (G0/G1)	[76]

↑ represent up-regulated protein levels and ↓ represent down-regulated protein levels. Akt: protein kinase B; AMPK: adenosine 5′-monophosphate (AMP)-activated protein kinase; AO: aldehyde oxidase; AP-1: activated protein-1; Apaf-1: apoptotic protease-activating factor 1; ASC: apoptosis-associated speck-like protein containing a CARD; Atg: anti-thymocyte globulin; Bak: Bcl-2 homologous antagonist/killer; Bax: apoptosis regulator BAX; BCL-2: B-cell lymphoma-2; Bcl-xL: B-cell lymphoma-extra large; BID: recombinant human BH3-interacting domain death agonist; BIR: biorepressor; BIRC: baculoviral IAP repeat-containing protein; BUN: blood urea nitrogen; c-FOS: c-fos proto-oncogene protein; c-JNK: c-Jun N-terminal kinase; CARD: caspase-recruitment domain-containing protein; Caspase: cysteinyl aspartate specific proteinase; CDC1: cell division control protein 2 homolog; CDC20: cell division cycle protein 20 homolog; CDC7: cell division cycle 7-related protein kinase; CDKs: cyclin-dependent kinases; CHK2: checkpoint kinase 2; CHOP: DNA damage-inducible transcript 3 protein; COX-2: prostaglandin–endoperoxide synthase; Cyto C: photosynthetic reaction center cytochrome c subunit; DDIT3: DNA damage-inducible transcript 3; DIABLO: diablo IAP-binding mitochondrial protein; E-cad: E-cadherin; E2F: transcription factor E2F; EGFR: epithelial growth factor receptor; EMT: epithelial–mesenchymal transition; ERK1/2: extracellular regulated protein kinases1/2; ERN1: endoplasmic reticulum to nucleus signaling 1; FADD: signal transducer and activator of transcription 3; GADD: growth arrest and DNA damage; GGT: gamma glutathione hydrolase 1 proenzyme; Glut: facilitative glucose transporter; IAP: inhibitor of apoptosis protein; ICMT: protein-S-isoprenylcysteine O-methyltransferase; IKKα: IKK-alpha; IL-1β: interleukin-1 beta; IL-6: interleukin-6; IκBα: inhibitor kappa B alpha; LC3: light chain 3 (microtubule-associated proteins light chain 3); LDH: lactate dehydrogenase; LMNA: prelamin-A/C; MAPK: mitogen-activated protein kinases; MCL-1: induced myeloid leukemia cell differentiation protein Mcl-1; MDA: malondialdehyde; MDM2: murine double minute 2; MEK: mitogen-activated extracellular signal-regulated kinase; MMP-2/9: matrix metalloproteinase 2/9; MMPs: matrix metalloproteinases; mTOR: mammalian target of rapamycin; MTX: methotrexate; N-cad: N-cadherin; NF-κB: nuclear factor kappa-B; NLRP3: nucleotide- binding oligomerization domain; NO: nitrous oxide; NOS2: nitric oxide synthase 2; PARP-1: poly (ADP-ribose) polymerase 1; PCNA: proliferating cell nuclear antigen; PD-L1: programmed cell death-ligand 1; PI3K: phosphoinositide 3-kinase; PKM2: pyruvate kinase isozyme typeM2; PPAP: prostatic acid phosphatase; PUMA: P53 up-regulated modulator of apoptosis; Raf: rapidly accelerated fibrosarcoma; Ras: Ras-like protein 1; RIP1: zinc metalloproteaser ip1; ROS: reactive oxygen species; SFN: stratifin (epithelial cell marker protein 1); Smad: Drosophila mothers against decapentaplegic protein; SP-1: specificity protein 1; STAT3: signal transducer and activator of transcription 3; TFEB: transcription factor EB; TLR4: toll-like receptor 4; TNF: tumor necrosis factor; TRAF2: TNF receptor-associated factor 2; TRAIL: TNF-related apoptosis-inducing ligand; TXNIP: thioredoxin-interactingprotein; UBC: ubiquitin C; VEGF: vascular endothelial growth factor; ZBTB17: zinc finger and BTB domain-containing protein 17; ZEB1: zinc finger E-box binding homeobox 1.

**Table 3:** Types and mechanisms of alleviating the toxic side effects of anti-tumor drugs with glycyrrhiza.

Disease	Experimental Model	Molding Dose	Compound	Therapeutic Dose	Mechanism	Ref.
Intestinal Toxicity	Cisplatin, Female Wistar Rats	10 mg/kg (i.p)	Glycyrrhetinic Acid	50–100 mg/kg (i.g)	↑GSH ↓NF-κB, ↓TNF-α, ↓Caspase-3/6/9	[84]
Intestinal Mucositis	5-Fluorouracil, BALB/c Mice	384 μmol/kg (i.p)	Isoliquiritigenin	117–384 μmol/kg (i.p)	↓PTGS2, ↓NOS2, ↓TNF-α, ↓NF-κB p65	[87]
Hepatotoxicity	MTX, Male Wistar Rats	20 mg/kg (i.p)	Glycyrrhetinic Acid	50–100 mg/kg (i.g)	↑GSH, ↑SOD, ↑GPx, ↑GST, ↑Bcl-2, ↑Nrf2, ↑HO-1, ↑PPARγ ↓MDA, ↓NO	[89]
Hepatotoxicity, Intestinal Toxicity	MTX, Male Wistar Rats	20 mg/kg (i.v)	Magnesium Isoglycyrrhizinate	9–18 mg/kg (i.v)	↑GSH, ↑SOD, ↑GPx, ↑Bax, ↑Caspase-3, ↑ZO-1, ↑cleaved-PARP ↓Bcl-2, ↓COX-2, ↓Cx43	[90]
Hepato-renal Damage	MTX, Female Wistar Albino Rats	20 mg/kg (i.p)	Glycyrrhiza Glabra Rhizome Extract	100–400 mg/kg (i.g)	↑IL-10, ↑dilatation, ↑hemorrhage, ↑congestion ↓MDA, ↓GSH, ↓SOD, ↓CAT, ↓peroxidase, ↓glutathione, ↓AST, ↓ALT, ↓BUN, ↓TNF-α, ↓IL-1β, ↓NF-kB, ↓IL-6, ↓IL-12, ↓Gapsase-3	[96]
Hepatocellular Damage	Cisplatin, Male Albino Rats	7 mg/kg (i.p)	*G. glabra* Extract	400 mg/kg (i.g)	↑SOD, ↑GSH ↓ALT, ↓AST, ↓ALP, ↓MDA, ↓MDA, ↓IL-1β, ↓Caspase-9, ↓NF-kB	[97]
Hepatotoxicity	Cisplatin, Male Wistar Rats	8 mg/kg (i.p)	Licorice	255–900 mg/kg (i.g)	Caspase-3, ↑HMGB1, ↑SOD, ↑ABCC2, ↑Bcl-2, ↑p53 ↓TNF-α, ↓IL-Iβ, ↓MDA, ↓CYP1A2, ↓CYP1A1, ↓ESR1, ↓TOP2A, ↓Cyp4a2, ↓ABCB1B	[98]
Hepatotoxicity	2% Acetaminophen, CSD Rats/Wister Rats/ICR Mice	100-500 mg/kg (i.p)	Glycyrrhizin	25 mg/kg (i.g)	↑GSH, ↑leucocyte, ↑K^+^, ↑CD4^+^/CD8^+^ ↓γ-GT, ↓Na^+^	[91]
Acute Liver Injury	LPS/GalN, WT, Nrf2^−/−^ in Male C57BL/6 Mice	WT: 10-30μg/kg; Nrf2^−/−^: 600-700mg/Kg (i.p)	Licochalcone A	50–100 mg/kg	↑GSH, ↑SOD, ↑Trx-1, ↑Nrf2, ↑HO-1, ↑P62, ↑Atg7, ↑Atg12, ↑Atg16, ↑Beclin-1, ↑Atg5, ↑Atg3, ↑LC3II, ↑AMPK, ↑TFEB ↓AST, ↓ALT, ↓IL-6, ↓IL-1β, ↓TNF-α, ↓MDA, ↓ROS, ↓JNK, ↓ERK, ↓P38, ↓IκBα, ↓NF-κ (P65), ↓TLR4, ↓Txnip, ↓NLRP3, ↓ASC,↓Mature-IL-1β, ↓cleaved-Caspase-1	[99]
Nephrotoxicity	Cisplatin, Male BALB/c Mice	30 mg/kg (i.p)	Glycyrrhizic Acid	GA: 25–100 mg/kg 18β-GA: 10–50 mg/kg (i.g)	↑catalase, ↑SOD, ↑GPx, ↑GSH/GSSG, ↑Nrf2, ↓BUN, ↓creatinine, ↓LDH, ↓HO-1, ↓TNF-α, ↓IL-1β, ↓IL-6, ↓HMGB1	[94]
Genotoxicity, Nephrotoxicity	Cisplatin, Male Swiss Albino Mice	7 mg/kg (i.p)	Glycyrrhizic Acid	75–150 mg/kg (i.g)	↑GR, ↑GST, ↑GPx ↓BUN, ↓creatinine, ↓LDH	[93]
Acute Kidney Injury	Cisplatin, Cell Model	Mptc:5-10µg/mLHK2:10µg/mL	Isoliquiritin	62.5 µM	↑Bcl-2/Bax, ↑Bcl-2, ↑SOD2, ↓Caspase-3, ↓ROS, ↓IL-6, ↓P p-65/p-65	[95]

↑ represent up-regulated protein levels and ↓ represent down-regulated protein levels. ABCB1B: ATP-binding cassette protein B1b; ABCC2: ATP-binding cassette sub-family C member 2; ALP: alkaline phosphatase; ALT: alanine aminotransferase; AST: aspartate transaminase; CAT: catalase; Cx43: connexin 43; CYP1A1: cytochrome P450 1A1/A2; Cyp4a2: cytochrome P450 4A2; ESR1: estrogen receptor; GPx: glutathione peroxidase; GR: gluathione reductase; GSSG: glutathione; HMGB1: high-mobility group box chromosomal protein 1; HO-1: heme oxygenase 1; MAD: max dimerization protein 1; PPARγ: peroxisome proliferator-activated receptor gamma; PTGS2: prostaglandin-endoperoxide synthase 2; SOD: superoxide dismutase; TOP2A: DNA topoisomerase 2-alpha: Trx-1: thioredoxin-1; ZO-1: zonula occludens protein 1; γ-GT: γ-glutamy transpeptidase.

**Table 4:** Healthcare products based on glycyrrhiza.

Product Name	Component Content (Per 100 g Content)	Drug Efficacy	Approval Number
Small gourd treasure brand ganoderma licorice tea	Crude polysaccharide 1.7 g Total flavonoids 0.32 g	Immunity enhancement	G20200250
Ji Zhentang brand astragalus and date licorice tablets	Total saponins 0.22 g		G20190149
Xinxi licorice tablet	Glycyrrhizic acid 3 g		G20190365
Jinglifeng brand herb Ganoderma licorice tea	Total saponins 0.3 g Total flavonoids 0.5 g		G20200313
Shaner brand licorice Prince ginseng capsule	Total saponins 1.4 g Crude polysaccharides 3.5 g		G20210131
Xia Fang brand licorice tablets	Glycyrrhizin 3.2 g		G20190335
Jiang Green brand licorice tablets	Glycyrrhizin 4.4 g		G20090146
Liyuan brand kudzu glycyrrhiza vitamin C tablets	Total flavone 4.4 g	Protection from chemical liver injury	G20200386
Qiansha brand sanqi pueraria licorice capsule	Total flavonoids 0.779 g Total saponins 1.4 g		G20100134
Huaxia Xianbao R officinale licorice capsule	Total saponins 0.4 g	Clear heat from throat	G20190414
Itai brand sweet licorice	Glycyrrhizin 0.0375 g		2003-0184
Moyin licorice Wumei lozenges	Glycyrrhizin 0.594 g		G20060562

## Data Availability

All data generated or analyzed during this study are included in this paper, and further inquiries can be directed to the corresponding author (E-mail: 20080011@nxmu.edu.cn, 20080017@nxmu.edu.cn).
